# Pituitary apoplexy following shoulder arthroplasty: a case report

**DOI:** 10.1186/1752-1947-5-284

**Published:** 2011-07-05

**Authors:** Savitha Madhusudhan, Thayur R Madhusudhan, Roger S Haslett, Amit Sinha

**Affiliations:** 1 St. Pauls Eye Unit, Royal Liverpool University Hospital, Liverpool L7 8XP, UK; 2 Department of Trauma and Orthopaedics, Glan Clwyd Hospital, Rhyl LL18 5UJ, UK; 3 Department of Ophthalmology, H M Stanley Hospital, St. Asaph LL17 0RS, UK

## Abstract

**Introduction:**

Pituitary apoplexy following a major surgical procedure is a catastrophic event and the diagnosis can be delayed in a previously asymptomatic patient. The decision on thromboprophylaxis in shoulder replacements in the absence of definite guidelines, rests on a careful clinical judgment.

**Case presentation:**

A previously healthy 62-year-old Caucasian male patient who underwent shoulder arthroplasty developed hyponatremia resistant to correction with saline replacement. The patient had a positive family history of deep vein thrombosis and pulmonary embolism and heparin thromboprophylaxis was considered on clinical grounds. The patient developed hyponatremia resistant to conventional treatment and later developed ocular localizing signs with oculomotor nerve palsy. The diagnosis was delayed due to other confounding factors in the immediate post-operative period. Subsequent workup confirmed a pituitary adenoma with features of pituitary insufficiency. The patient was managed successfully on conservative lines with a multidisciplinary approach.

**Conclusions:**

A high index of suspicion is required in the presence of isolated post-operative hyponatremia resistant to medical correction. A central cause, in particular pituitary adenoma, should be suspected early. Thromboprophylaxis in shoulder replacements needs careful consideration as it may be a contributory factor in precipitating this life-threatening condition.

## Introduction

Pituitary apoplexy resulting from an acute hemorrhage or infarction of the pituitary gland usually occurs in a macroadenoma. The rapid increase in tumor volume results in an abrupt onset of a variable combination of neurological symptoms and signs including headache, vomiting, ocular nerve palsies, visual field defects, visual acuity impairment, Horner's syndrome, stroke, meningism, stupor and coma as well as endocrine dysfunction.

## Case presentation

A 62-year-old Caucasian male patient was admitted for right total shoulder replacement for an arthritic shoulder. He was on treatment with non-steroidal anti-inflammatory medication for pain relief, a thiazide diuretic, calcium channel blocker and beta blockers for hypertension, and low dose aspirin for cardioprotection. There was a family history of deep vein thrombosis and pulmonary embolism. He was a non-smoker and did not consume alcohol. His systemic examination, routine pre-operative blood investigations and ECG were normal. He was accepted for the elective procedure under the ASA 2 category.

The operation was performed under general anesthesia in the beach chair position. There were no intra-operative complications. Following the surgery, the patient was prescribed opioid analgesics for pain control and a low molecular weight heparin for thromboprophylaxis, in view of the strong family history of deep vein thrombosis. For comfort the operated arm was supported in a sling, with gentle assisted exercises.

On the second post-operative day the patient was mobilizing well but was drowsy and confused; GCS was 15 with no localizing signs and his vital parameters were normal. Post-operative blood parameters were normal except for low sodium (Table 1). Normal saline infusion was prescribed for correction of hyponatremia.

**Table 1:** Blood biochemistry values (all values in mmol/L)

Date	Sodium	Potassium	Urea	Creatinine	Glucose	CRP	LFT
pre-op	137	4.1	7.0	84	4-8	3 U	Normal

2^st ^post-op day	127	4.4	4.4	94	4.9	34 U	

5^th ^post op day	128	5.1	6.8	126			

7^th ^post op day	132	5.1	8.5	118			

4 weeks post op	138	3.8	5.9	114	6.3		

On the third post-operative day, the patient had a high temperature and the surgical wound appeared inflamed but there was no discharge. Blood samples were sterile on culture. Heparin thromboprophylaxis was discontinued and mechanical thromboprophylactic measures were instituted.

Over the next 48 hours, the patient complained of increasing bilateral frontal headaches with onset of binocular diplopia on the fifth post-operative day which was closely followed by increased urinary output, worsening confusion and drowsiness. The patient was transferred to the acute medical unit for further evaluation and management.

In the acute medical unit, the patient continued to be drowsy but oriented. His vital observations were normal. The GCS was 13 with no neck stiffness or photophobia. His speech was normal with good swallowing reflexes. There were no motor or sensory deficits in any of the four limbs. His gait was normal, Rhomberg's sign was negative and there were no other localizing signs. His bladder and bowel functions were normal.

**Figure 1:**
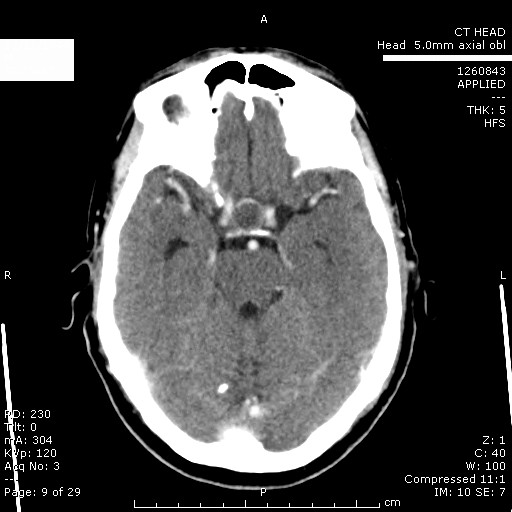
**Coronal CT scan showing the pituitary infarct**.

Ophthalmologic evaluation at this stage recorded visual acuities of 6/5 in the right eye and 6/9 in the left eye. The pupils were equal and reacting normally to light with brisk direct and consensual reflexes. There was no anisocoria. Fundus examination revealed normal optic discs with well-defined margins and normal retinal vasculature. A complete ptosis of the right upper eyelid and restriction of adduction, elevation and depression in the right eye suggested a pupil-sparing right complete third nerve palsy. A computed tomography (CT) brain scan (Figure 1) was requested to rule out any compressive pathology causing this nerve palsy, which revealed a low attenuation signal in the pituitary fossa suggestive of either a thrombosed aneurysm or a bleed into the pituitary gland. Pituitary function tests and an endocrinologist's opinion were requested. Pituitary profile and synacthen stimulation tests suggested pan-hypopituitarism (Table 2, Table 3). It is important to note that recent onset central adrenal insufficiency (secondary to apoplexy), often has an adequate serum cortisol response to ACTH. He was prescribed hydrocortisone and thyroxine replacement therapy.

**Table 2:** Hormonal values: 5^th ^post op day and at 1 year follow-up

Date	Free T4	TSH	LH	FSH	Prolac-tin	Corti-sol	Free testos-terone	Testos-terone	ACTH	SHBG
5 days post op	6 pm/L	0.36 mu	0.3 u	0.7 u	17 mu/l	30 nm/L	.002 nm/L	0.1 nm/L	17 ng/L	30 nm/L

4 weeks post op	12 pm/L	1.3 mu/L								16 nm/L

**Table 3:** Synacthen test

5 days post op	0 mins - 321 nmol/L 65 mins - 214 nmol/L
12 weeks post op	0 mins - 452 nmol/L 65 mins - 299 nmol/L

In the next 24 hours, he complained of reduced vision in his left eye; visual acuities now measured 6/5 in the right eye and 6/12 in the left eye; color desaturation in the temporal hemifields was noted, using a red target. A confrontation field test suggested superior bitemporal quandrantanopia; more accurate examination with automated perimetry was not possible, given the patient's condition. His optic discs continued to be normal. Repeat blood tests were normal except for low sodium and low hemoglobin levels (Table 1). Thiazides were discontinued and oral ferrous sulfate supplementation was initiated.

He was optimized medically and transferred to a tertiary center for further evaluation. An MRI scan of his brain further confirmed that the pituitary stalk was markedly deviated to the right with an enhancing area in the pituitary fossa, suggesting an adenoma. There was no extension of the pituitary into the suprasellar cistern and the chiasm was not compressed. His internal carotid artery anatomy was normal. He was managed further on conservative lines and discharged on hydrocortisone and thyroxine supplements, and testosterone replacement therapy.

On subsequent review, visual acuities had recovered to 6/5 in both eyes; color vision was normal; diplopia had resolved with fully restored extra-ocular movements. There was no ptosis, indicating good recovery from the third nerve palsy. A repeat MRI scan of our patient's brain at three months showed a small residual adenoma in the right pituitary fossa with the gland displaced to the left. He was discharged from active care on successful recovery and is being followed up on elective outpatient medical, ophthalmology and shoulder clinics.

## Discussion

The clinical presentation of pituitary apoplexy can be variable. Although in retrospect our patient had some of the classical findings - headache, altered mental status, oculomotor nerve palsy and visual field loss and persistent hyponatremia, resistant to medical correction - in the presence of confounding factors including the use of thiazide diuretics, expected transient fluid-electrolyte imbalance following general anesthesia and surgical blood loss [1], and the suspicion of sepsis in the immediate post-operative period, the diagnosis was delayed until the evolution of ophthalmic signs. These signs prompted radiological imaging and a definite diagnosis. Pituitary apoplexy presenting as hyponatremia secondary to hormone deficient adrenal insufficiency, following orthopedic surgery is documented, [2] but requires a high index of suspicion for investigations to be directed towards an early diagnosis.

Cranial nerve involvement in pituitary tumor and apoplexy has been well described. Compression of the oculomotor nerve in its intra-cavernous course as the gland swells and impinges on the cavernous sinus, commonly gives rise to mydriasis, limitation of eye movement and ptosis in that sequence [3]. The superficially located pupillomotor fibers are easily prone to compression from space-occupying lesions, while microvascular disease affecting the vasa nervosum in the main nerve trunk usually spares the pupillary fibers. Pupillary involvement provides an important clue in differentiating medical and surgical causes, although pupillary sparing does not always exclude a compressive lesion. Pituitary apoplexy can cause a sudden increase in the size of a pre-existing pituitary tumor and temporary impingement on the optic chiasm giving rise to bitemporal hemianopia and impaired vision.

Despite various medications and medical interventions, head trauma and pregnancy have been described as being causative of pituitary apoplexy [4-6] along with spontaneous bleeding into a pituitary neoplasm, when associated precipitating factors are not always easily identifiable [7]. Following major surgeries, hemodilution, hypotension, anti-coagulation and stress have all been implicated as risk factors. Pre-operatively our patient was asymptomatic and there were no signs of intra-cranial mass effect or pituitary dysfunction from the pituitary adenoma. Therefore further investigations were not carried out. With no definite identifiable factors responsible for his post-operative intra-tumor bleed, we believe heparin treatment might have been contributory in precipitating clinically symptomatic pituitary apoplexy. There are other reported cases of anticoagulation therapy precipitating pituitary tumor apoplexy [8].

Deep vein thrombosis and pulmonary embolism are recognized complications of total hip and knee arthroplasty [9] and definite guidelines for prevention and treatment of these complications exist for effective peri-operative management. However no guidelines exist for elective shoulder replacements. Several case reports and the occurrence of deep vein thrombosis and pulmonary embolism following shoulder replacements have been described in the published literature [10-13]. Due to the paucity of available evidence for thromboprophylaxis in shoulder replacements, the operating surgeon in many situations has to use his clinical judgment. In our patient, there was a strong family history of deep vein thrombosis and pulmonary embolism and low molecular weight heparin thromboprophylaxis was instituted. Until definite evidence and guidelines are available, heparin therapy in shoulder replacements will need careful consideration.

## Conclusion

Pituitary apoplexy as a cause of persistent hyponatremia resistant to medical correction is possible following shoulder replacement surgery. The presentation may be atypical particularly in the presence of various confounding factors in the peri-operative period. A multidisciplinary team input is required, for better management and for preventing life-threatening complications. Conservative management with hormone replacement is successful in patients even with delayed presentation and diagnosis.

## Consent

Written informed consent was obtained from the patient for publication of this case report and any accompanying images. A copy of the written consent is available for review by the Editor-in-Chief of this journal.

## Competing interests

The authors declare that they have no competing interests.

## Authors' contributions

SM is the principal author and was involved in the collection of data, review of the literature, and preparation of the manuscript. TRM was involved in the collection of relevant literature and proof read the manuscript. RH and AS were senior authors and were actively involved in patient care and proof read the manuscript. All authors were actively involved in direct patient care and have read and approved the manuscript.
